# Reticulon 4a/NogoA locates to regions of high membrane curvature and may have a role in nuclear envelope growth

**DOI:** 10.1016/j.jsb.2007.08.005

**Published:** 2007-11

**Authors:** Elena Kiseleva, Ksenia N. Morozova, Gia K. Voeltz, Terrence D. Allen, Martin W. Goldberg

**Affiliations:** a Laboratory of Morphology and Function of Cell Structure, Institute of Cytology and Genetics, Russian Academy of Science, Novosibirsk, 630090, Russia; b Department of Molecular, Cellular, and Developmental Biology, University of Colorado, Boulder, CO 80309, USA; c Paterson Institute for Cancer Research, Christie Hospital NHS Trust, Wilmslow Road, Manchester M20 4BX, UK; d School of Biological and Biomedical Sciences, Durham University, South Road, Durham, DH1 3LE, UK

**Keywords:** Reticulon 4a, NogoA nuclear envelope, Scanning electron microscopy

## Abstract

Reticulon 4a (Rtn4a) is a membrane protein that shapes tubules of the endoplasmic reticulum (ER). The ER is attached to the nuclear envelope (NE) during interphase and has a role in post mitotic/meiotic NE reassembly. We speculated that Rtn4a has a role in NE dynamics. Using immuno-electron microscopy we found that Rtn4a is located at junctions between membranes in the cytoplasm, and between cytoplasmic membranes and the outer nuclear membrane in growing *Xenopus* oocyte nuclei. We found that during NE assembly in *Xenopus* egg extracts, Rtn4a localises to the edges of membranes that are flattening onto the chromatin. These results demonstrate that Rtn4a locates to regions of high membrane curvature in the ER and the assembling NE. Previously it was shown that incubation of egg extracts with antibodies against Rtn4a caused ER to form into large vesicles instead of tubules. To test whether Rtn4a contributes to NE assembly, we added the same Rtn4a antibody to nuclear assembly reactions. Chromatin was enclosed by membranes containing nuclear pore complexes, but nuclei did not grow. Instead large sacs of ER membranes attached to, but did not integrate into the NE. It is possible therefore that Rtn4a may have a role in NE assembly.

## 1 Introduction

The nuclear envelope (NE) encloses the nucleus (Hetzer et al., 2005; Goldberg, 2004) and consists of two parallel sheets of membrane connected at nuclear pore complexes (NPCs). The outer membrane is continuous with the rough endoplasmic reticulum (ER). During mitosis and meiosis in higher eukaryotes the NE is dismantled. The lamina and NPCs are solubilised and the membranes disperse into the cytoplasm by retraction into the ER (Ellenberg et al., 1997; Yang et al., 1997) or by vesiculation (Vigers and Lohka, 1991).

During telophase the NE is reassembled around chromosomes in a multistage process, involving accumulation of different membrane populations and NE and NPC proteins. Evidence from *Xenopus* egg extracts suggest that there are least two vesicle populations (Vigers and Lohka, 1991; Macaulay and Forbes, 1996; Drummond et al., 1999) which fuse to form tubules and sheets (Wiese et al., 1997; Goldberg et al., 1992; Hetzer et al., 2001) which enclose the chromatin. Fusion of nuclear membranes requires hydrolysis of GTP (Macaulay and Forbes, 1996) by Ran (Hetzer et al., 2000; Zhang and Clarke, 2000; Zhang et al., 2002) and also the p97–UFD1–NPL4 complex (Hetzer et al., 2001). The mechanisms of targeting and fusion are unknown. NPCs are apparent after a few minutes in egg extracts (Goldberg et al., 1992), but NPC proteins accumulate in a temporal order in culture cells (Bodoor et al., 1999) as do structural intermediates in the NPC assembly process (Goldberg et al., 1997; Kiseleva et al., 2001).

During S-phase the NE grows as the DNA content in the nucleus is increased (Winey et al., 1997). The NE also grows during oogenesis. Although interphase NE growth is often considered separately from telophase, there are common features. Conceptually, once the chromatin is enclosed in telophase, there is no obvious difference between the subsequent growth phase and interphase growth. Like enclosure, growth requires the AAA-ATPase p97, but instead of UFD1 and NPL4 it is complexed with p47 (Hetzer et al., 2001), suggesting the mechanisms are related but distinct.

Rtn4a/NogoA (hereafter referred to as Rtn4a) is a member of reticulon family and is one of three splice variants of the RTN4 gene (Oertle and Schwab, 2003). It has attracted much interest recently because of its inhibitory role in neurite outgrowth (Prinjha et al., 2000; Yan et al., 2006). Rtn4a has also been shown to localise to the ER (van de Velde et al., 1994) but is restricted to the tubular network and excluded from the peripheral ER sheets and NE (Voeltz et al., 2006). Rtn4a was shown to be required for formation of ER tubules from sheets and vesicles (Voeltz et al., 2006) and it was suggested that reticulons could induce and stabilise the high curvature of the membrane required to maintain tubules. It is possible that the unusual topography of the reticulons, with their long putative transmembrane domains, could induce curvature when clustered.

Rtn4a does not appear to be concentrated at the NE during interphase (Voeltz et al., 2006) possibly because the NE membrane bilayers consist of flat sheets with low membrane curvature. However, nuclear assembly involves considerable reorganisation of membrane topology. It is thought that ER tubules, shaped by Rtn4a (Voeltz et al., 2006), feed into the NE (Ellenberg et al., 1997) and vesicles may also contribute (Vigers and Lohka, 1991; Liu et al., 2003; Prunuske et al., 2005). These vesicles and tubules have to be converted to a large flattened double sheet during NE assembly. Recently, it was shown that Rtn4a may have an essential role in NE disassembly in *C. elegans* (Audhya et al., 2007). Therefore, we decided to test if Rtn4a could have a role in NE formation, both during interphase and telophase. We used a high resolution surface imaging technique, field emission in-lens scanning electron microscopy (feiSEM^1^) to look at the structure of interphase growing NEs during *Xenopus* oogenesis and in telophase *in vitro*. We show that highly curved membrane regions of the forming or growing NEs preferentially contain Rtn4a. Such regions include the junctions between apparently fusing vesicles and the edges of flattening membranes. An antibody against Rtn4a was also shown to affect NE assembly. We therefore suggest that Rtn4a could have a role in NE formation and growth.

## 2 Results

The ER connects to the NE in interphase and has been implicated in the post mitotic assembly of the NE (Ellenberg et al., 1997; Yang et al., 1997; Mattaj, 2004). Much of the interphase ER forms a tubular network which appears to feed into the NE during reassembly (Ellenberg et al., 1997; Hetzer et al., 2001). Rtn4a has been found to be involved in shaping the ER into tubules in specific regions (Voeltz et al., 2006). Therefore, we speculated that if the ER needs to be in a tubular form to contribute to NE growth and assembly then Rtn4a may have a role. First we asked whether Rtn4a is associated with membranes that are contributing to NE growth, and then we investigated whether Rtn4a might be required for NE assembly.

### 2.1 Rtn4a is located at inter-membrane junctions between cytoplasmic vesicles near the NE

During oogenesis in *Xenopus,* oocytes are arrested in pre-prophase when the nucleus grows to over 100 μM diameter. The NE must expand by the addition of membranes and assembly of lamina and NPCs. We previously observed, both in thin section TEM of whole oocytes and isolated nuclei and in feiSEM of isolated NEs, that growing stage III oocytes have more extraneous ER-like membranes associated with the NE than mature stage VI (Morozova and Kiseleva, 2006).

**Fig. 1:**
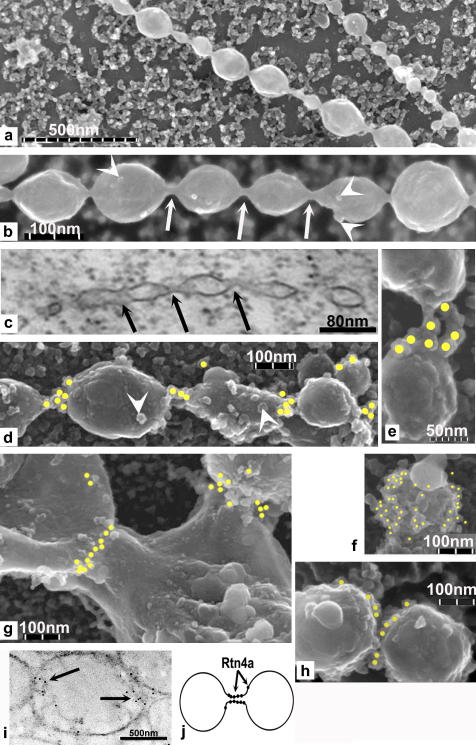
Membrane structures associated with stage III oocyte NEs have Rtn4a at their membrane-membrane junction. (a and b) feiSEM of strings of vesicle-like structures at the NE showing 20 nm bridges (arrows) and ribosomes (arrowheads). (c) TEM of strings of vesicle-like structure at the NE showing bridges (arrows). (d and e) Rtn4a immuno-gold labelling of strings of vesicle-like structures at the connecting tubule. (f) Some vesicle-like structures have dense labelling of Rtn4a more evenly distributed over the surface. (g and h) Localisation of Rtn4a at the junction between larger membrane structures. (i) TEM sections of oocyte cytoplasmic membranes immuno-gold labelled for Rtn4a (arrows). (j) Our interpretation of these structures and the localisation of Rtn4a. Circles mark the position of immuno-gold particles determined by superimposing a simultaneously obtained backscatter electron image (see Section 4.1).

FeiSEM analysis of the extraneous membranes localised near the NE showed that many were present as structures that look like long lines of inter-connected vesicles (Fig. 1a). We are not certain of the origin of these structures but their surface has an ER-like appearance, with ribosome-like particles on the surface (Fig. 1b and d, arrowheads). The vesicle-like structures are joined together by a short thin tubular connection of ∼20 nm diameter (Fig. 1b, white arrows). The same structures were also observed in TEM thin sections of whole oocytes, showing that they are not artifacts of NE isolation or feiSEM specimen preparation (Fig. 1c). We have also used different fixation methods (see Section 4.1). In TEM sections (Fig. 1c) the 20 nm diameter inter-connecting tubes are continuous with the vesicle membranes and therefore appear to be membrane bridges between vesicle-like structures.

Anti-Rtn4a immuno-gold labelling with a previously characterised affinity purified anti *Xenopus* Rtn4a antibody (Voeltz et al., 2006) showed that Rtn4a was present on the surface of vesicles (Fig. 1f). In the inter-connected vesicle structures, Rtn4a labelling was concentrated at the junctions between connected membrane structures where the membrane bridge is located (Fig. 1d and e). Inter-connected larger membrane structures were also observed (Fig. 1g and h) where Rtn4a was located at the junction between them. Immuno-gold labelling of TEM sections of whole oocytes also shows localisation of Rtn4a at the contact point of adjacent vesicles. (Fig. 1i). Rtn4a therefore appears to locate to the inter-connections between membrane structures (Fig. 1j). These results show that Rtn4a locates to specific regions on NE associated cytoplasmic membranes.

### 2.2 Rtn4a is present on membranes attached to growing NEs

**Fig. 2:**
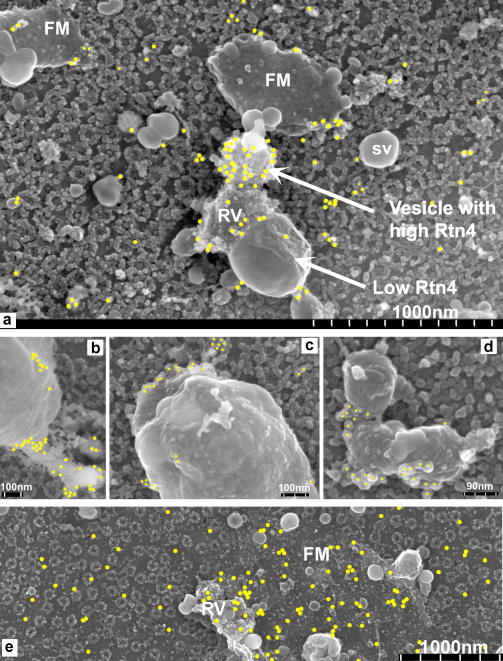
Immuno-gold labelling shows Rtn4a localises to NE associated membranes. (a) FeiSEM image of the surface of a stage III oocyte NE showing rough ribosome containing vesicles (RV), smooth vesicles (SV) and flattened membrane (FM). The position of anti-Rtn4a immuno-gold particles detected using a backscatter detector is indicated by circles. (b–d) Rtn4a localises to regions of contact between larger membrane structures and the ONM. (e) ER protein, ribophorin, has a more even distribution over flattened membranes, vesicles and the outer nuclear membrane.

We isolated NEs from stage III oocytes and immuno-gold labelled them for Rtn4a. There was some labelling of the ONM (Fig. 2a) but membrane structures associated with the NE were heavily labelled and in some cases, in a specific pattern. Fig. 2a is an image of the surface of a stage III NE showing NPCs, rough (ribosome-containing) vesicles (RV), smooth (ribosome-free) vesicles (SV), and rough membranes that appear to be flattening onto the ONM (FM). We see that there is labelling of the ONM and vesicles are labelled to a varying degree. Smooth vesicles are not labelled, whereas rough ER type vesicles are. This shows that Rtn4a is associated with some but not all membranes that are associated with the ONM of a growing NE.

### 2.3 Rtn4a localises to the junction between the ONM and membranes attached to it

Membrane structures could be seen attached to the ONM. Rtn4a appeared to be located near the point of contact between the membrane structures and the ONM (Fig. 2b–d). Therefore, it appears that Rtn4a accumulates both at junctions between ER-like membranes and between NE associated membranes that are attached to the ONM. NEs labelled with an antibody (CEL5C) to the ER protein ribophorin (Drummond et al., 1999) showed a more even distribution over flattened membranes, some vesicles and the outer nuclear membrane (Fig. 2e).

### 2.4 Rtn4a localises to the edges of flattened membranes

**Fig. 3:**
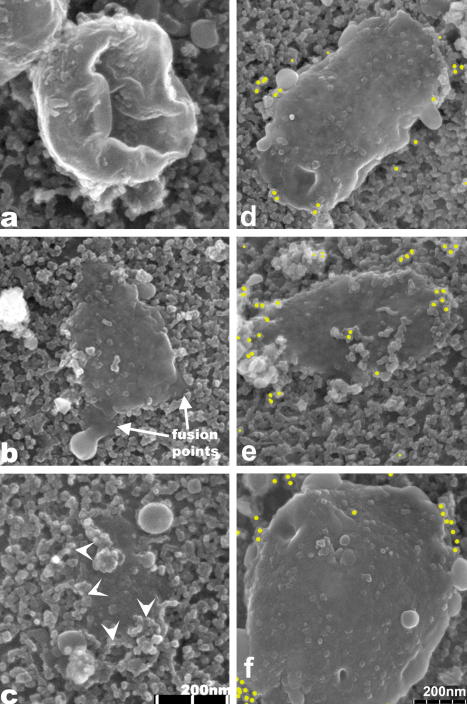
Flattened membranes at the ONM of stage III oocyte. (a–c) Attached membranes with different inferred degrees of flattening: (a) is attached but not flattened; (b) is flattened with a few apparent points of fusion (arrows) and (c) is more integrated in the ONM and NPCs (arrowheads) are present around the edges (d–f) Immuno-gold labelling of Rtn4a in flattened membranes showing edge position of Rtn4a (circles). Scale bar in (c) refers to (a–c), scale bar in (f) refers to (d–f).

**Fig. 4:**
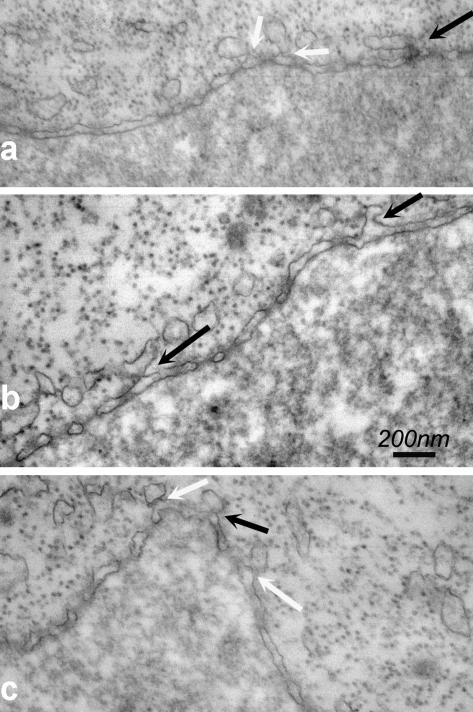
Thin section TEM of NE with cytoplasmic membranes attached. Membranes may be continuous with the ONM (black arrows) or attached but apparently not continuous (white arrows).

Some of the observed NE associated membranes had the appearance of collapsed spheres (Fig. 3a) whereas many are flattened (Fig. 3b–f). It is not always clear from feiSEM images whether such membranes are fused to the ONM or simply lying on top, so thin section TEM was carried out and showed continuity between the ONM and overlying flattened membrane structures (Fig. 4, black arrow). Points of contact between the ONM and cytoplasmic membrane structures were also observed (Fig. 4, white arrows). Potentially corresponding images were seen by feiSEM, in which the flattened membrane structures appear continuous with the NPC-containing ONM (Fig. 3b, arrows).

**Fig. 5:**
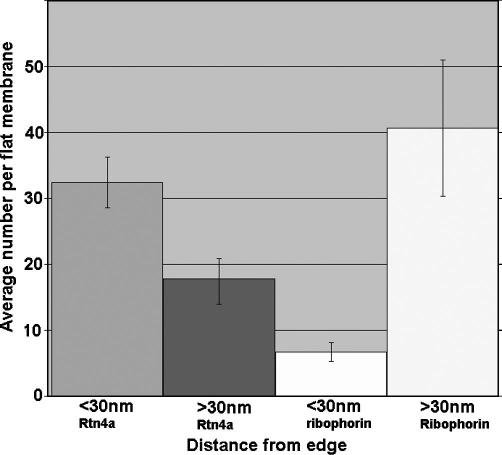
Rtn4a locates to the edges of flattened membranes. The average number of gold particles on each flattened membrane structure that are located less than 30 nm from the edge was compared to those located more than 30 nm from its edge. This was compared to the distribution of the ER protein, ribophorin. Bars represent standard error of the mean.

Using immuno-gold labelling, we observed that Rtn4a appeared to locate preferentially around the edges of the flattened membranes (Fig. 3d–f). This was quantified by counting the number of gold labels that were within 30 nm of the edge compared to greater than 30 nm from the edge (Fig. 5), which gave a ratio of approximately 2:1, for edge compared to the interior. This was in contrast to the ER protein, ribophorin, which was distributed more away from the edge (Fig. 5). The preferred edge location suggests that Rtn4a tends to locate or accumulate at regions with the highest curvature. We conclude that Rtn4a marks the highly curved edges of flattened sheets of presumed ER membrane attached to the ONM.

### 2.5 Rtn4a is present at the edges of membranes that are flattening onto the chromatin

**Fig. 6:**
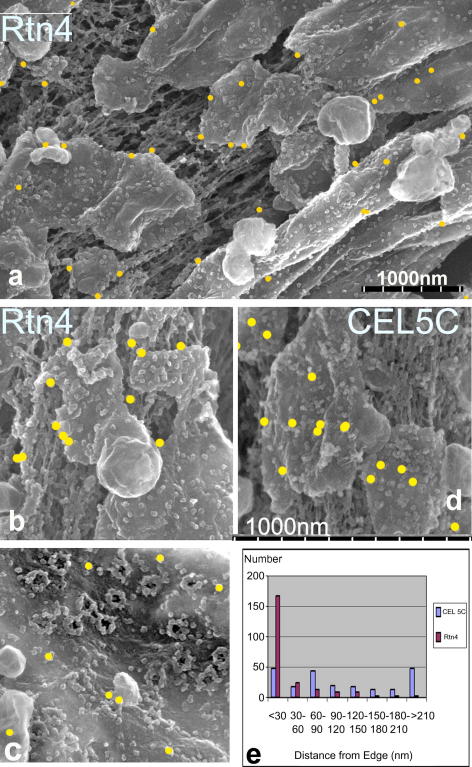
Membranes flattening onto chromatin during the early (a, b and d) stages of NE assembly in *Xenopus* egg extracts and labelled with antibodies to Rtn4a (a–c) or ribophorin (antibody CEL5C), marked by circles (d). (c) Fully assembled NE labelled for Rtn4a. (d) Quantification of gold labels for the two antibodies plotted as a distance from the edge. Scale bar in (d) refers to (b–d).

During telophase, membranes also flatten onto chromatin (Goldberg et al., 1992; Macaulay and Forbes, 1996). We wanted to test if the edge location of Rtn4a that we observed in growing oocyte NEs also occurred in chromatin bound flattening membranes. To do this, we used a cell free system from *Xenopus* eggs which can be used to assemble nuclei *in vitro* (Goldberg and Allen, 1993; Lohka and Masui, 1984). Nuclei were isolated from assembly reactions, fixed and immuno-gold labelled for Rtn4a. At early stages (2–5 min) vesicles bind to the chromatin and flatten (Goldberg et al., 1992; Wiese et al., 1997). In such vesicles, Rtn4a localises preferentially around the edges (Fig. 6a and b), compared to a general ER protein, ribophorin (antibody CEL5C—Drummond et al., 1999), which was more randomly distributed (Fig. 6d). To show this formally we counted the distribution of gold particles located within 30 nm of the vesicle edge, or further away in 30 nm increments, using images from three separate experiments (Fig. 6e).This shows that Rtn4a, compared to ribophorin, is preferentially located near the membrane edge. Therefore, as in growing oocytes, Rtn4a preferentially locates to the edges of flattening membranes. At later stages of assembly when the chromatin is enclosed, and there are no longer any highly curved membrane edges (except at the nuclear pores), the Rtn4a distribution on the NE appeared random and low level (Fig. 6c). This is consistent with Rtn4a’s preference for curved membranes (Voeltz et al., 2006). Although there are highly curved membranes in the nuclear pores we see no Rtn4a labelling there. The high membrane curvature at the NPC might be maintained by nucleoporins which could exclude the accumulation of Rtn4a, or it is possible that Rtn4a is present but not detected by the antibody due to epitope masking.

### 2.6 Disrupting Rtn4a prevents NE growth

Although Rtn4a is not preferentially located to the NE during interphase (Voeltz et al., 2006), our results in stage III oocytes show that it does locate to membranes that are attached to the interphase NE and suggested the possibility that it may be involved in NE assembly or growth. Therefore, we wanted to investigate if Rtn4a might be required for NE assembly. We incubated egg extracts with a previously characterised anti *Xenopus* Rtn4a antibody directed against the cytoplasmic facing N-terminus (Voeltz et al., 2006) which is specific to Rtn4a and not present in other Rtn4 spliced variants or other reticulon proteins.

**Fig. 7:**
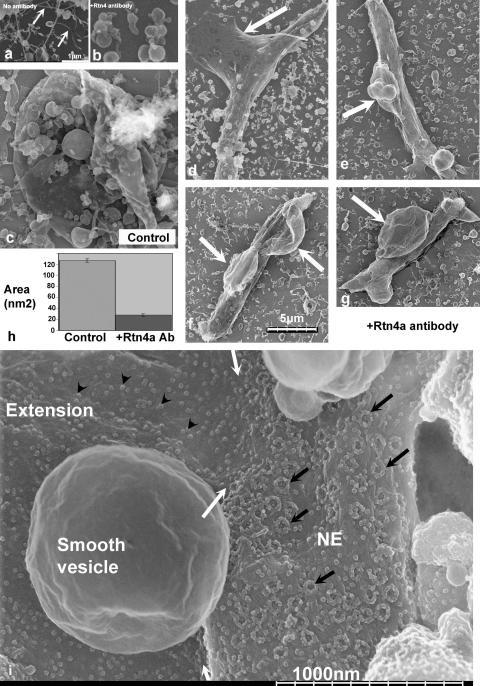
Rtn4a antibody perturbs NE growth in *Xenopus* egg extract. The antibody prevents the usual formation of ER tubules (a, arrows), instead forming large vesicles (b). In control nuclear assembly reactions (c) nuclei are large and roughly round, whereas in the presence of the antibody they remain sperm shaped with large membrane extensions (arrows) (d–g). (h) Nuclear growth is significantly retarded by the Rtn4a antibody as shown by quantification of the two-dimensional area of nuclei observed by feiSEM. (i) High magnification image of nucleus assembled in the presence of Rtn4a antibody, showing NPCs (black arrows) assembled on the sperm chromatin shaped region and the NPC-free ER-like extension with ribosomes (arrowheads). White arrows indicate the junction between the chromatin bound NE and the ER-like extension. Scale bar in (a) applies to (a and b). Scale bar in (f) applies to (c–g).

This antibody inhibits the formation of ER tubules in similar egg extracts (Voeltz et al., 2006) and likewise we found that it inhibited ER tubule formation. Instead, ER membranes assembled into large vesicular structures (Fig. 7b) rather than tubules as seen in the no-antibody control reactions (Fig. 7a). These large membrane structures are ER-derived because they have ribosomes on their surface. This shows that the anti-Rtn4a antibody had a dominant effect on the formation of ER tubules.

To determine if the Rtn4a antibody affected NE assembly, the same egg extracts were incubated with the antibody (see Section 4.1) or with buffer or with an irrelevant antibody, before adding sperm chromatin to initiate nuclear assembly. Controls showed rapid binding, fusion and flattening of vesicles onto chromatin, following by chromatin decondensation, enclosure and NE growth to form large roughly spherical nuclei >10 microns in diameter, as expected (Goldberg et al., 1997; Wiese et al., 1997 and Fig. 7c). In the presence of the Rtn4a antibody we found that nuclei did assemble with an apparently completely enclosed unbroken NE (Fig. 7d–g) that had apparently normal NPCs (Fig. 7i, black arrows). However, the chromatin failed to decondense and remained as a dense sperm-shaped object surrounded by NE (Fig. 7d–g). To quantify this apparent NE growth defect, we measured the two-dimensional area occupied by each nucleus (excluding attached membranes, see below) as viewed from above by feiSEM (Fig. 7h). Nuclei in Rtn4a antibody-inhibited reactions had ∼80% reduced area compared to controls, confirming the growth defect.

There were in addition, large (several microns) membrane structures extending from the nucleus (Fig. 7d–g, arrows). The extensions are clearly rough ER-like and contain ribosomes (Fig. 7i, arrowheads). There is a sharp demarcation between the chromatin attached membrane which contains NPCs and the membrane extensions which contain ribosomes but not NPCs (Fig. 7i, white arrows). The extensions are clearly continuous with the NPC-containing NE. Therefore, it appears that perturbing Rtn4a does not prevent enclosure of the chromatin by NE. However, the chromatin and NE do not expand despite the attachment of large ER-like sacs to the ONM. These sacs are similar to the large ER vesicle formed in the cytosol in the presence of the antibody, except they are attached to the NE.

Antibodies and reagents against other NE and ER proteins do not have such an affect on nuclear assembly or ER tubulation. Addition of wheat germ agglutinin, which binds to certain nucleoporins (Hanover et al., 1987), to extracts results in nuclei without NPCs but it has no effect on ER tubulation and does not result in membrane extensions (Goldberg et al., 1997). Addition of anti-nucleoporin antibodies (Mab414 and QE5) (unpublished results) or depletion of extracts with antibodies to specific nucleoporins, Nup214 (Walther et al., 2002) or Nup153 (Walther et al., 2001), also affected NPC structure but again does not affect the ER or NE membranes. Depletion of lamin B3 resulted in small spherical nuclei with normal NPCs but had no obvious effect on ER tubules or membrane extensions (Goldberg et al., 1995). The CEL5C antibody against the ER protein ribophorin (Drummond et al., 1999) also did not affect tubulation or membrane extensions when added to extracts (unpublished result). Similar experiments were also done (Voeltz et al., 2006) using antibodies to IP3R and TRAPα which also did not affect ER tubule formation. Therefore, we believe that the growth defects and extensions are a specific effect of the anti-Rtn4a antibody used in this study.

## 3 Discussion

We have found that Rtn4a is localised to junctions between membrane structures and at the edges of flattened membranes associated with growing NEs both in oocytes and in nuclei assembling *in vitro*. This is the first direct evidence to support the proposal that Rtn4a locates to regions of high curvature (Voeltz et al., 2006). It is a unique localisation that has not been seen for other NE or ER proteins. We believe this localisation may be driven by Rtn4a itself rather than by interacting with other proteins because high level over-expression of Rtn4a in COS cells (Voeltz et al., 2006) and the related Rtn1 in yeast (De Craene et al., 2006) does not change the proteins’ localisations and therefore is unlikely to rely on other titratable factors. The location of Rtn4a at the junction between cytoplasmic membranes and NE membranes suggested to us the possibility that Rtn4a may also have a role in NE assembly.

Addition of the Rtn4a antibody to nuclear assembly reactions allowed NE formation in egg extracts but chromatin remained condensed and the NE did not expand. Therefore, the antibody has a dominant effect on the growth phase of NE assembly but not on the initial enclosure and NPC assembly. As Rtn4a is an integral membrane protein that appears to be involved in shaping membranes (Voeltz et al., 2006) we suggest that the effect of the antibody is to perturb nuclear membrane dynamics and assembly, as shown previously for the ER (Voeltz et al., 2006).

One possible speculation for the localisation of Rtn4a at membrane–membrane junctions in oocytes is that it may take part in fusion or stabilisation of membrane curvature during fusion. Inter-membrane fusion involves transitory extremes of curvature involving membrane stalks between vesicles (Smeijers et al., 2006; Yang and Huang, 2002). Rtn4a could be involved in stalk formation or stabilisation in certain NE membranes. We do not know if these membranes in oocytes are actively fusing membranes or if they are more stable or intermediate structures. Rtn4a could be involved in stabilising the conformation of these junctions by maintaining the high membrane curvature to facilitate membrane flow into the NE.

Our *in vitro* antibody inhibition experiments suggest that ER-like membranes can attach to the NE when Rtn4a is perturbed but the NE fails to grow. When Rtn4a is perturbed the ER membranes are converted to large sacs, which can attach to the NE but fail to contribute to NE expansion. This suggests the possibility that the organisation of the interface between the NE and ER, as observed in oocytes, may be important for NE growth. Therefore we speculate that a possible function of localising Rtn4a to the inter-membrane junctions between ONM and ER is to maintain a particular interfacial organisation which contributes to the movement of membrane into the NE. Our results argue against a model in which membranes simply diffuse into the NE during assembly and growth (Ellenberg et al., 1997) because the NE fails to grow when Rtn4a is perturbed despite the attachment of ER membranes to the NE.

Rtn4a is not only located at the inter-membrane junctions but also throughout the tubular ER, where it appears to be involved in maintaining the tubularity (Voeltz et al. 2006; Fig. 7). Therefore, the tubular nature of the ER may be essential for its function in providing membrane for NE growth, at least in egg extracts.

The Rtn4a antibody does not inhibit the initial formation of NE around chromatin. This NE appears normal: it is flattened, fused and contains NPCs. Because it contains NPCs we can conclude that the contributing membranes carried the integral membrane nucleoporins such as POM121 and gp210 enabling NPC formation. This suggests that Rtn4a may only be required during the growth phase and that membranes that contribute to initial enclosure and NPC formation (Yang et al., 1997; Drummond et al., 1999) are not perturbed by the antibody.

We have also found that Rtn4a locates specifically to the edges of flattening membranes in growing and assembling NE in both *in vivo* and *in vitro* experiments. As Rtn4a has been implicated in the formation of highly curved membrane regions, it is possible to speculate that Rtn4a is important for formation of flattened sheets, by stabilisation of the high curvature at the edge regions. However, this may be a non essential function in the initial stages of NE assembly, which occur in the presence of the antibody.

## 4 Conclusions

We have shown for the first time that Rtn4a partitions, within a single membrane structure, to the region of highest curvature, supporting the model that reticulons preferentially locate to curved membranes (Voeltz et al., 2006) and have a function related to membrane curvature. We have also observed that it locates to membrane junctions and other highly curved regions of membranes that may be involved in NE growth. Concordantly, an Rtn4a antibody perturbs NE growth. We therefore hypothesise that Rtn4a may have a role in maintaining functional ER–NE junctions during NE growth and/or the ER must be tubular to contribute to NE growth.

### 4.1 Materials and methods

#### 4.1.1 Isolation and fixation *Xenopus* oocyte for feiSEM

Stage III and VI NEs were isolated in 5:1 buffer, spread, fixed in 2% glutaraldehyde, 0.2% tannic acid, 10 mM Tris–HCl (pH 7.4) and 0.3 mM MgCl_2_ and processed for feiSEM as described previously (Kiseleva et al. 2004). In some experiments tannic acid, which helps preserve protein filament (Maupin and Pollard, 1983) was omitted, as it can effect membrane preservation (MWG unpublished), but the same membrane structures were observed. Samples were coated with chromium using a Cressington 308R with additional cryo-pump or an Edwards Auto306 with cryo-pump to a nominal thickness of 2 nm. They were viewed using a Hitachi S-5200 feiSEM at 10 kV accelerating voltage.

#### 4.1.2 Fixation *Xenopus* oocyte for TEM

Oocytes were fixed (2.5% glutaraldehyde, 0,1M Hepes) for 1 h, then washed twice in 0.1 M Hepes buffer and postfixed 1 h in 1% OsO_4_ in ddH_2_O at 4 °C, stained 2 h in 1% aqueous uranyl acetate, washed in water, dehydrated through ethanol series and embedded in Agar-100 (Agar Scientific, UK). Sections were stained with Lead citrate and viewed with Leo 910 (Germany) TEM at 80 kV.

#### 4.1.3 Immuno-TEM

Stage III oocytes were fixed in 4% formaldehyde (TAAB Labs) in Ringers (111 mM Nacl, 1.9 mM KCl, 1.1 mM CaCl_2_, 2.4 mM NaHCO_3_, pH 7.0) overnight at 4 °C, washed three times in Ringers, stain in 2% uranyl acetate in Ringers for 2 h at 4 °C, then washed in water twice, dehydrated in 30% ethanol, then further dehydrated by freeze substitution as follows through a series of ethanol (30% at 4 °C for 1 h, 50% at −20 °C for 1 h, 70% at −20 °C for 1 h, 95% at −20 °C for 2 h), embedded to LR Gold (Agar) at −20 °C and polymerised in gelatin capsules under UV light at −15 °C for 48 h. Ultrathin sections were cut and attached to nickel grids then incubated with 1% BSA in PBS for 30 min, washed in PBS, incubated with 1:100 dilution anti-Rtn4a rabbit antibody for 1 h, washed in PBS and incubated with a goat anti-rabbit secondary antibody conjugated to 10 nm colloidal gold (Amersham) for 1 h and washed with PBS. Sections were stained with lead citrate for 2 min.

#### 4.1.4 Immuno-gold labelling and feiSEM imaging

Affinity purified polyclonal antibody (40 M stock) against N-terminal domain of *Xenopus* Rtn4a (Voeltz et al., 2006) was diluted 1:100 with PBS. Nuclei were isolated and fixed for 20 min in 3.7% formaldehyde, 5:1 buffer, washed three times with PBS, incubated in PBS, 1% BSA 30 min, washed in PBS, and incubated 1–3 h primary antibody, PBS. Samples were washed three times in PBS, and incubated 1 h with 10 nm gold-conjugated secondary goat anti-rabbit antibody (Amersham Corp.). As negative controls, we used gold-conjugated secondary antibody diluted 1:100 in PBS. All samples were then washed three times in PBS then in 10 mM Tris–HCl and processed for feiSEM as above. Both secondary electron images (for structure) and backscatter electron images (for gold label position) were collected simultaneously. Using Adobe Photoshop, the backscatter image was superimposed onto the secondary image as a separate layer. The positions of the gold particles were marked with a yellow dot and then the backscatter image removed.

#### 4.1.5 *In vitro* nuclear assembly

Egg extracts were prepared as described previously (Goldberg et al., 1997). To perturb Rtn4a, extract was incubated with 4 μM affinity purified *Xenopus* Rtn4a antibody (Voeltz et al., 2006) on ice for 20 min then 1000 sperm chromatin per microlitre was added and the extract warmed to room temperature for 2–60 min for assembly. In controls an equal volume of either buffer or irrelevant antibody was added, which had no detectable effect on nuclear assembly. Extracts were resuspended in Membrane Wash Buffer (MWB: 250 mM sucrose, 50 mM KCl, 2.5 mM MgCl_2_, 10 mM Hepes, pH 7.4) and centrifuged at 1000*g* at 4 °C for 10 min onto silicon chips (Agar Scientific Ltd) and immersed in Membrane Fix (150 mM sucrose, 1 mM MgCl_2_, 80 mM Pipes–KOH, pH 6.8, 2% paraformaldehyde, 0.25% glutaraldehyde) for 10 min and processed for feiSEM as previously described (Goldberg et al., 1997). Samples were imaged at 3 kV accelerating voltage.

#### 4.1.6 Antibody labelling of *in vitro* nuclei

Extract (5 μl) was resuspended in MWB and centrifuged at 1000*g* at 4 °C for 10 min onto silicon chips (Agar Scientific, UK). Chips were immersed in Membrane Fix without glutaraldehyde for 10 min, washed in PBS, immersed in PBS +100 mM glycine 10 min, washed in PBS, immersed in 1% fish skin gelatine (Sigma) for 1 h, then 1 μM primary antibody for 1 h, washed 3 times 5 min in PBS, incubated with 1:50 dilution anti-rabbit secondary antibody conjugated to 10 nm gold (Amersham) for 1 h, washed 3 times 5 min and 1 times 15 min in PBS and fixed in Membrane fix with 1% glutaraldehyde. Samples were then processed for feiSEM as previously described (Goldberg et al., 1997).

## Figures and Tables

**Supplementary Figure 1:**
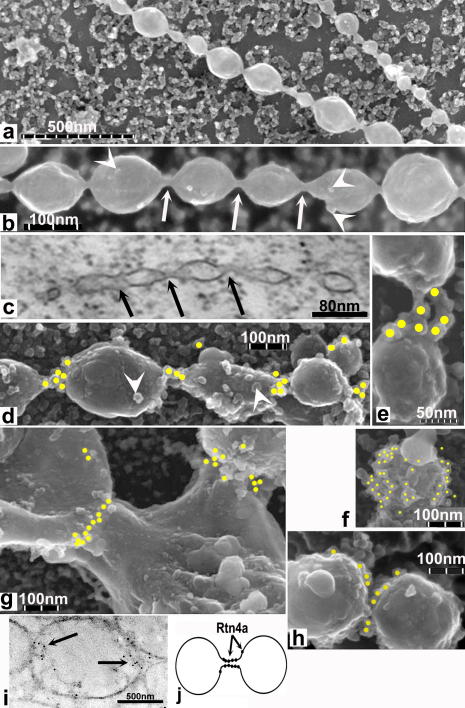


**Supplementary Figure 2:**
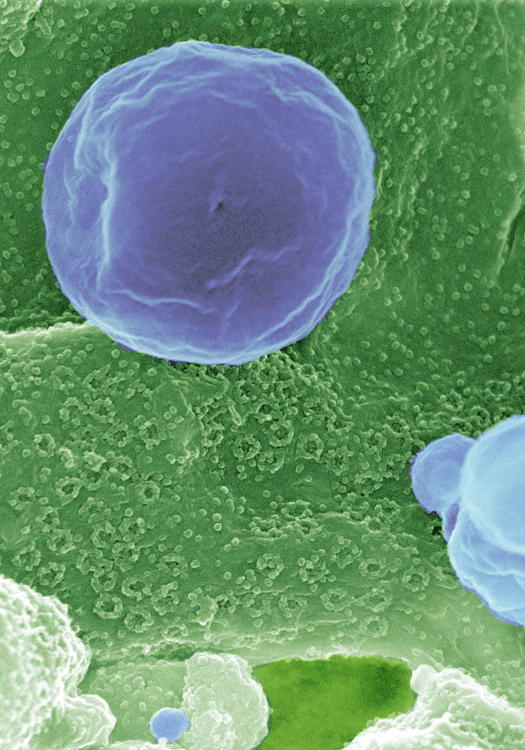


**Supplementary Figure 3:**
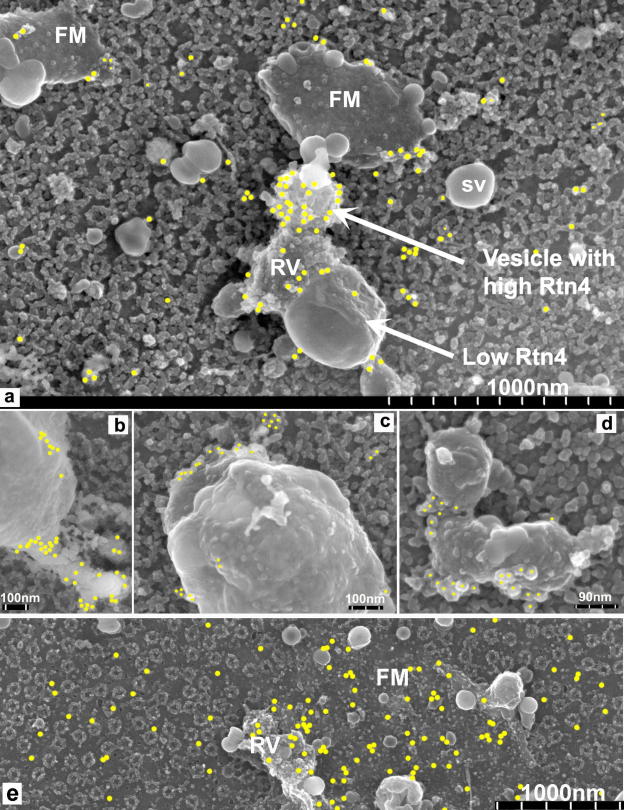


**Supplementary Figure 4:**
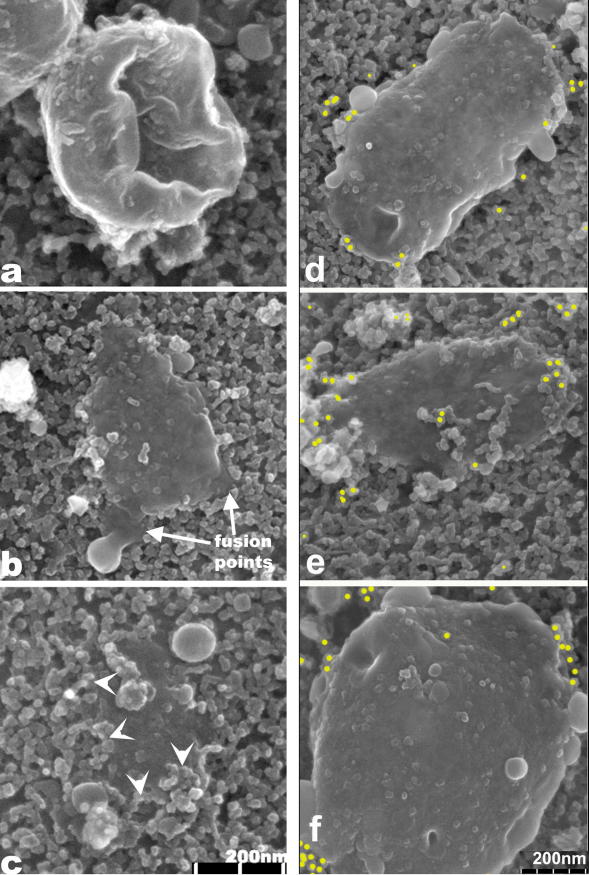


**Supplementary Figure 5:**
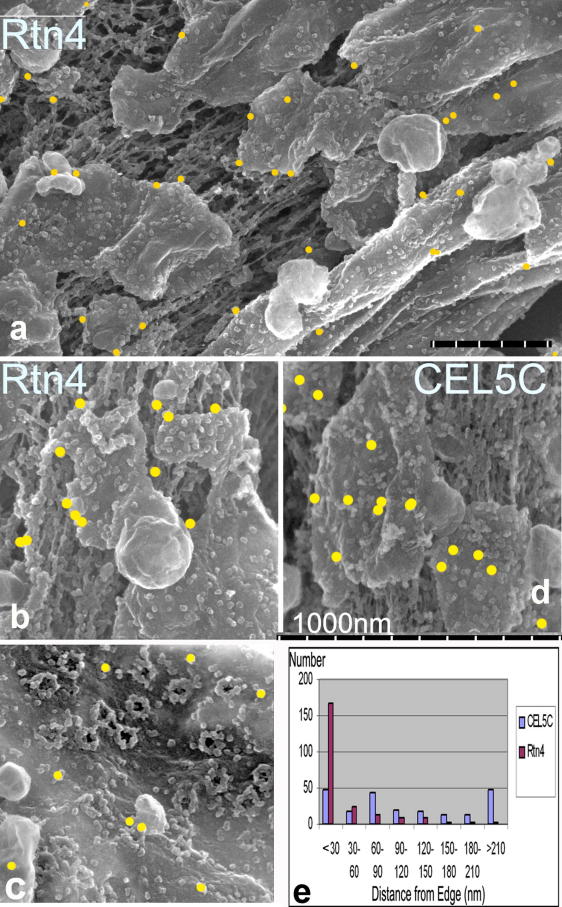


**Supplementary Figure 6:**
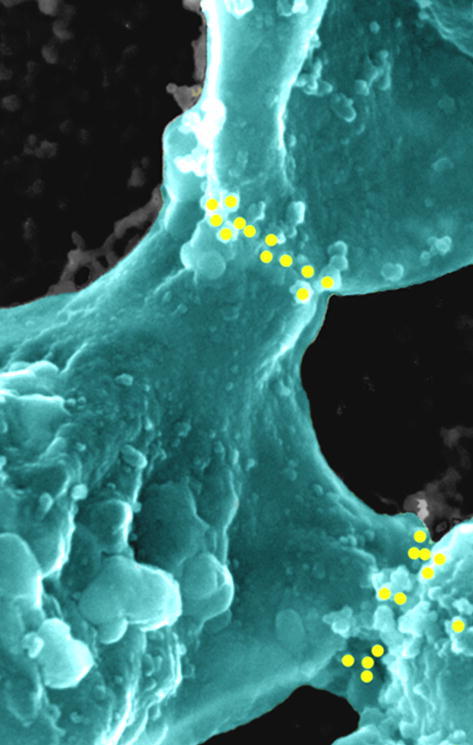


**Supplementary Figure 7:**
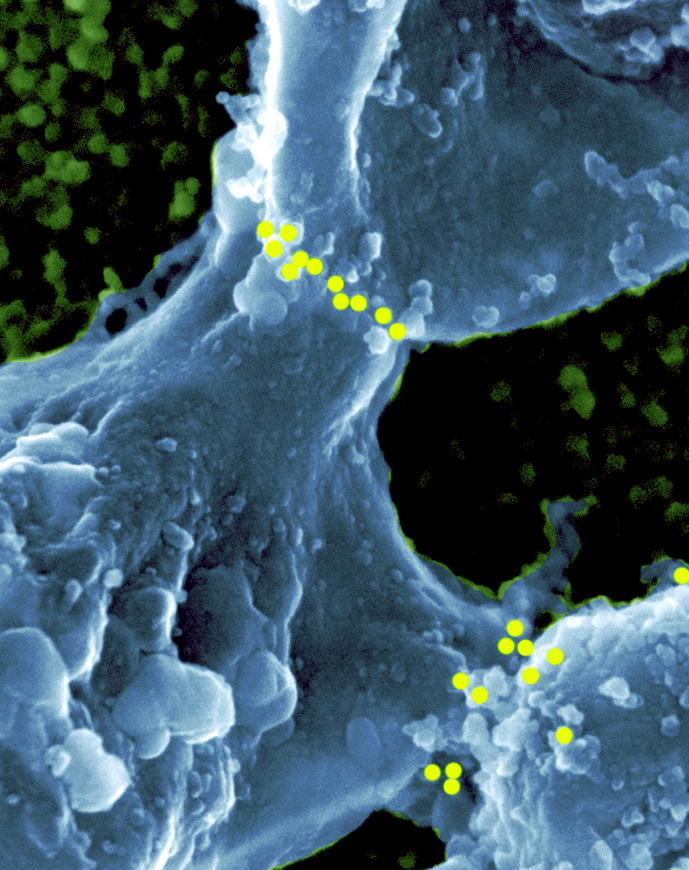


**Supplementary Figure 8:**
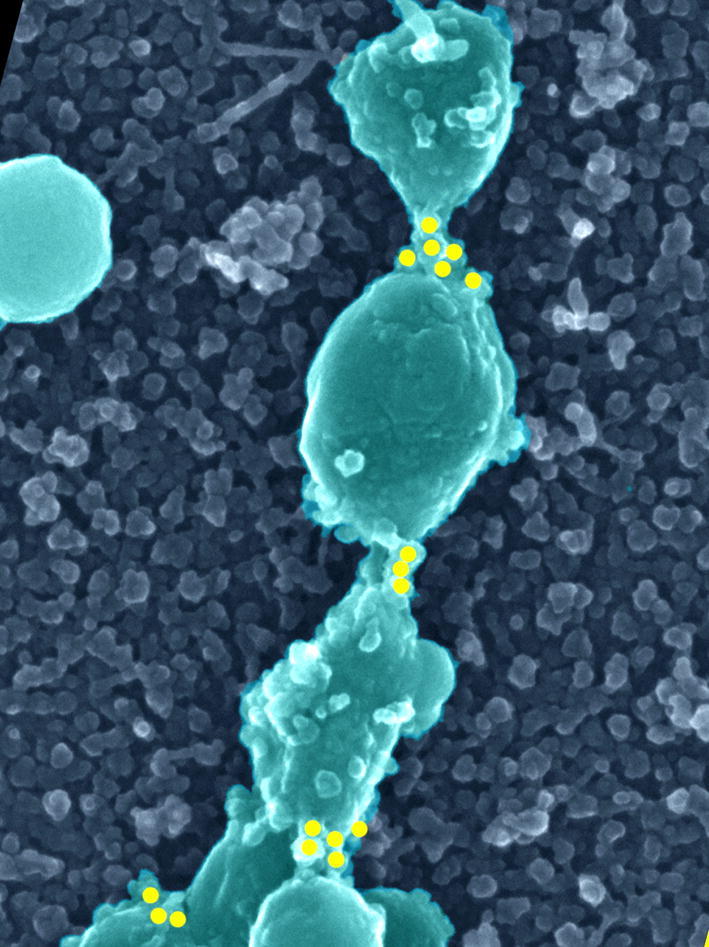


**Supplementary Figure 9:**
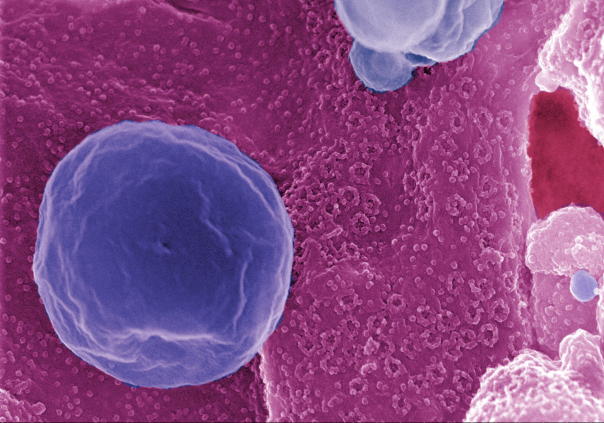


**Supplementary Figure 10:**
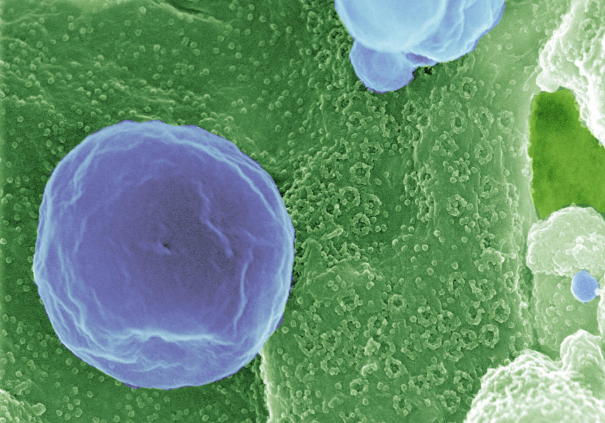


## Footnotes

1 *Abbreviations used:* feiSEM, field emission scanning electron microscopy; ER, endoplasmic reticulum; NE, nuclear envelope; Rtn4, areticulon 4a; TEM, transmission electron microscopy; NPC, nuclear pore complex; ONM, outer nuclear membrane.
